# Supplementary material for: Atomically resolved phase transition of fullerene cations solvated in helium droplets
Source: Nat Commun. 2016 Nov 22;7:13550. doi: 10.1038/ncomms13550 (PMC5121423; doi:10.1038/ncomms13550)
Supplement: Supplementary Information — Supplementary Figures 1-4, Supplementary Methods and Supplementary References [file ncomms13550-s1.pdf]

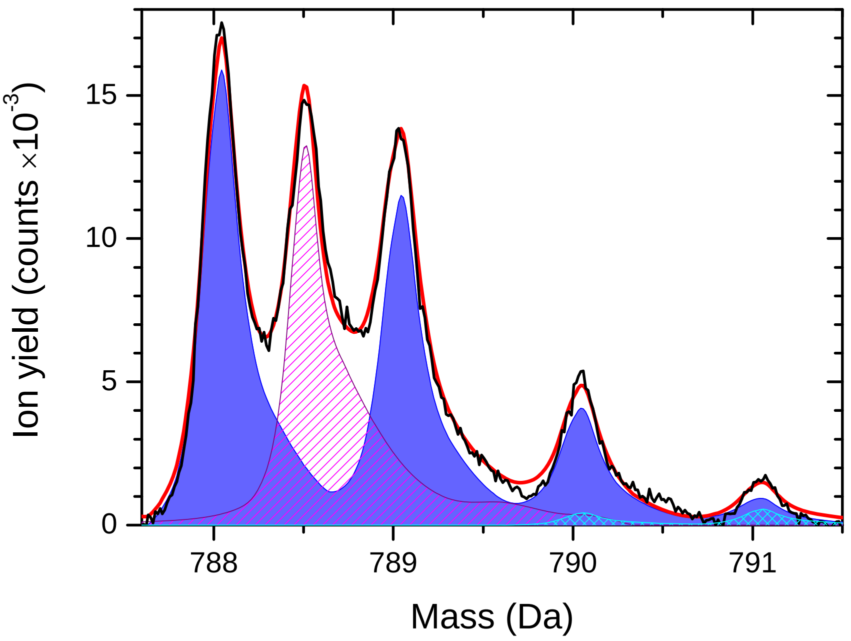

Supplementary Figure 1: Assignment of different ionic contributions to the mass spectrum around $He_{17}C_{60}^{+}$

Section of a mass spectrum taken at a laser wavelength of 963 nm of C_{60} doped helium droplets. The measured spectrum (solid black line) is fitted by the program IsotopeFit [1] and the result of this fit is shown as a solid red line. The main contributions in this mass spectrum are assigned to $He_{17}C_{60}^{+}$ (filled blue curve) and He_{197}^{+} (hatched magenta curve) and a small contribution of the ions $He_{13}H_2OC_{60}^{+}$ and $He_{13}H_3OC_{60}^{+}$ (hatched cyan curve).

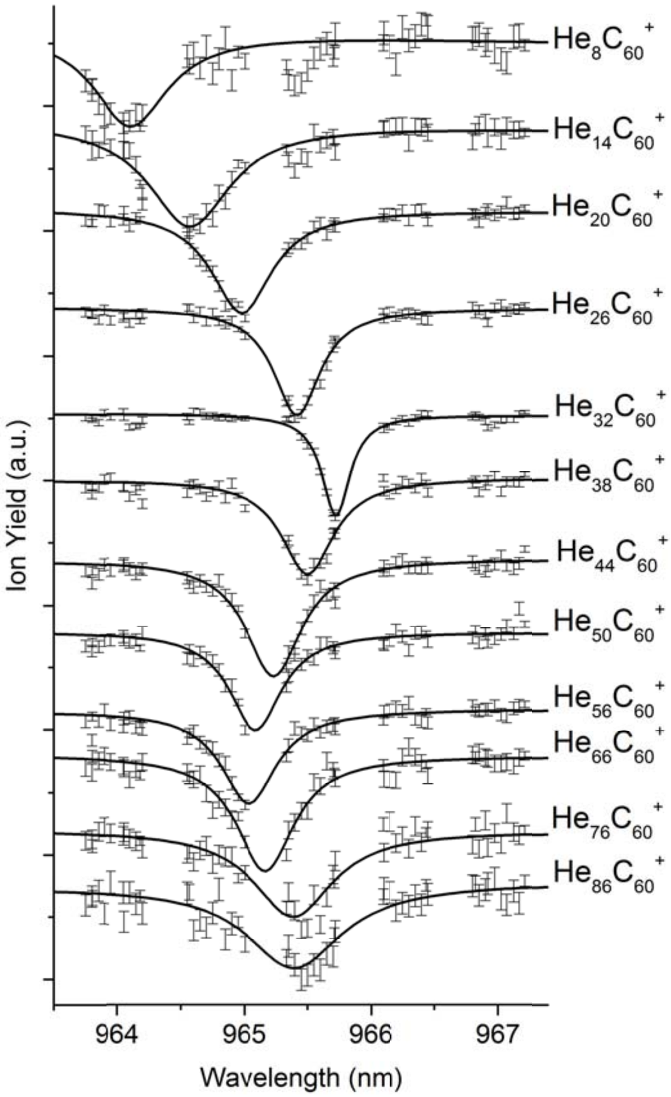

Supplementary Figure 2: Ion signal depletion for selected $He_nC_{60}^+$ ions around 964 nm

Wavelength scans near 964 nm for selected different cluster sizes. Photoabsorption depletes the ion signal to minima at different wavelength positions with a line width (full width at half maximum) of about 0.2 nm (for $He_{32}C_{60}^+$) to 0.6 nm ($He_{14}C_{60}^+$). The solid lines represent Lorentzian fits to the data points. Error bars indicate s.d.

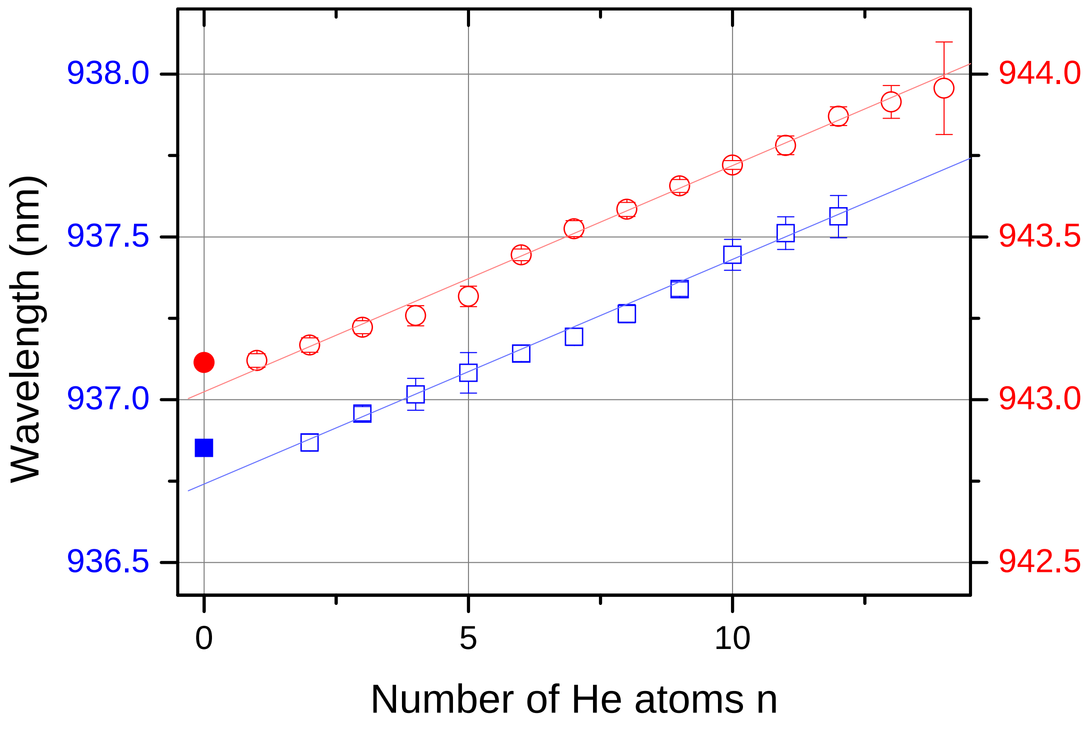

Supplementary Figure 3: Measured absorption wavelengths near 937 nm and 943 nm

Center positions for the absorption spectra of $He_nC_{60}^+$ around 937 nm (blue open squares, left y-axis) and 943 nm (red open circles, right y-axis) plotted as a function of n, the number of helium ad-atoms on the fullerene ion surface. The absorption wavelengths (corrected to vacuum) that were obtained for the bare C_{60}^+ ion by the group of Maier [2] are indicated by the bold symbols. For both curves the red-shift is again 0.07 nm per He atom. As in the case of the two strong resonances the wavelength shift from the bare C_{60}^+ to one attached He atom is smaller than 0.02 nm as mentioned in Ref. [2].

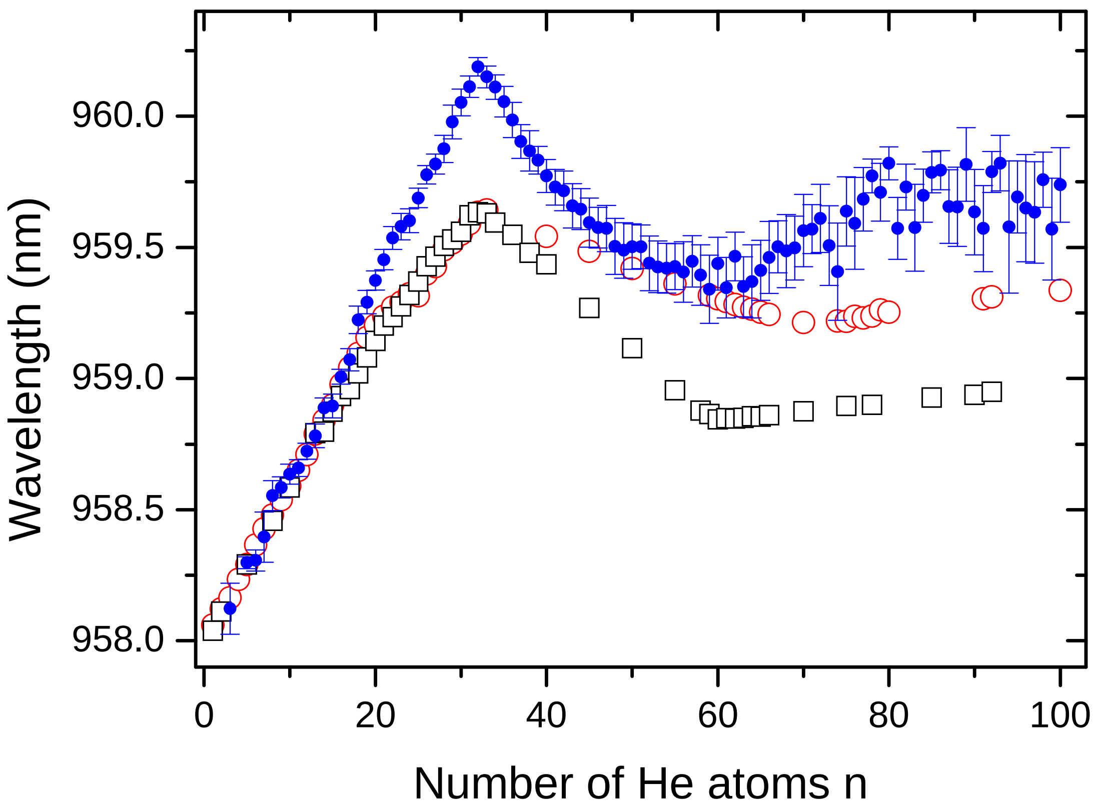

Supplementary Figure 4: Experimental and calculated absorption wavelengths

Calculated absorption wavelengths for $C_{60}^{+}He_n$ as a function of He atoms attached near 958 nm (open symbols) in comparison to the measured data (solid symbols). The error bars indicate the statistical uncertainties of the center of a Lorentzian profile fitted to the ion signal depletions shown in Fig. 3 of the main text and Supplementary Fig. 2. Open circles neglect and open squares include quantum effects. The latter were renormalized by a factor of 1.0008.

Supplementary Methods

2.1 Polarizability of the ground state of C_{60}^{+}

To calculate the integral $\langle \Psi_0 | \mu_z | \Psi_i \rangle$, we expand the Slater determinants of Ψ_0 and Ψ_i and apply the dipole operator, which is a sum of one-electron operators. The only non-vanishing terms are those in which the matrix elements in Ψ_0 and in Ψ_i only differ in one spin-orbital. Let the two determinants Ψ_0 and Ψ_i differ in spin-orbitals p and q, then

$$\langle \Psi_0 | \mu_z | \Psi_i \rangle = (-1)^{k_p - k_q} \langle p | z | q \rangle \quad \text{(S1)}$$

where k_p and k_q are, respectively, the position numbers of spin-orbitals p and q appearing in the Slater determinants.

As the angular parts of p and q are spherical harmonics, and noticing that $z = R_s \cos\theta = R_s \sqrt{\frac{4\pi}{3}} Y_1^0$, the integral $\langle \Psi_0 | z | \Psi_i \rangle$ eventually consists of integrals of products of three spherical harmonics $\langle Y_l^m | Y_1^0 | Y_{l'}^{m'} \rangle$. The integral can be analytically calculated:

$$\langle p | z | q \rangle \equiv (l_m | l'_{m'}) \equiv R_s \sqrt{\frac{4\pi}{3}} \langle Y_l^m | Y_1^0 | Y_{l'}^{m'} \rangle \quad \text{(S2)}$$

$$= R_s \sqrt{(2l+1)(2l'+1)} \begin{pmatrix} l & 1 & l' \\ 0 & 0 & 0 \end{pmatrix} \begin{pmatrix} l & 1 & l' \\ m & 0 & m' \end{pmatrix} \quad \text{(S3)}$$

The selection rules for non-vanishing integrals are $|l - l'| \le 1$ and $m = -m'$.

Therefore, we only need to consider the following singly-excited Slater determinants to calculate the polarizability of the ground state of the molecule, which are:

(i) The first excited state, $4_0 \to 5_0$ excitation $(\Delta l = 1; m = m' = 0)$ with one open shell:

$$\Psi_1 = \Psi_{4\to5} = \left| \ldots 4_0 5_0 \overline{5_0} \right| \quad \text{(S4)}$$

In this notation, only orbitals involved in the transition are indicated. Note that there are nine degenerate first excited states differing in the magnetic quantum number m corresponding to $l=4$. However, only the state with 4_0 gives non-vanishing coupling with the 5_0 state in Ψ_0 (being $\left(4_0 \mid 5_0\right)$), due to the above-mentioned selection rules. The contribution from the coupling with Ψ_1 to the ground state polarizability is

$$\alpha_0^1 = 2e^2 \frac{\left|\langle \Psi_0 \mid z \mid \Psi_1 \rangle\right|^2}{E_5 - E_4} = 2e^2 \frac{\left(4_0 \mid 5_0\right)^2}{E_5 - E_4} \quad \text{(S5)}$$

(ii) The $5_0 \to 6_0$ excitation $\left(\Delta l = 1; m = m' = 0\right)$ with one open shell:

$$\Psi_{5\to 6} = \left|\ldots 4_0 \overline{4_0} 6_0\right| \quad \text{(S6)}$$

The corresponding contribution to the ground state polarizability is

$$\alpha_0^{5_0\to 6_0} = 2e^2 \frac{\left(5_0 \mid 6_0\right)^2}{E_6 - E_5} \quad \text{(S7)}$$

(iii) The $4_m \to 5_{-m}$ excitation $\left(\Delta l = 1; m = -m' = \pm 3, \pm 4\right)$ with three open shells. This configuration has two doublets:

$$\Psi^a_{4_m\to 5_{-m}} = \frac{1}{\sqrt{2}}\left(\left|\ldots 4_m 5_0 \overline{5_{-m}}\right| - \left|\ldots 4_m \overline{5_0} 5_{-m}\right|\right) \quad \text{(S8)}$$

$$\Psi^b_{4_m\to 5_{-m}} = \frac{1}{\sqrt{6}}\left(\left|\ldots 4_m 5_0 \overline{5_{-m}}\right| + \left|\ldots 4_m \overline{5_0} 5_{-m}\right| - 2\left|\ldots \overline{4_m} 5_0 5_{-m}\right|\right) \quad \text{(S9)}$$

The corresponding contribution to the ground state polarizability is

$$\alpha_0^{4_m\to 5_{-m}} = 8e^2 \sum_{m=3}^{4} \frac{\left(4_m \mid 5_m\right)^2}{E_5 - E_4} \quad \text{(S10)}$$

(iv) The $5_m \to 6_{-m}$ excitation $\left(\Delta l = 1; m = -m' = \pm 1, \pm 2\right)$ with three open shells. This configuration has two doublets:

$$\Psi^{a}_{5_m \to 6_{-m}} = \frac{1}{\sqrt{2}}\left(\left|\ldots 5_0 5_m \overline{6_{-m}}\right| - \left|\ldots 5_0 \overline{5_m} 6_{-m}\right|\right) \tag{S11}$$

$$\Psi^{b}_{5_m \to 6_{-m}} = \frac{1}{\sqrt{6}}\left(\left|\ldots 5_0 5_m \overline{6_{-m}}\right| + \left|\ldots 5_0 \overline{5_m} 6_{-m}\right| - 2\left|\ldots \overline{5_0} 5_m 6_{-m}\right|\right) \tag{S12}$$

The corresponding contribution to the ground state polarizability is

$$\alpha_0^{5_m \to 6_{-m}} = 8e^2 \sum_{m=1}^{2} \frac{\left(5_m \,|\, 6_m\right)^2}{E_6 - E_5} \tag{S13}$$

Collecting all these contributions, the polarizability of the ground state of C_{60}^{+} is

$$\alpha_0 = 2e^2 \frac{\left(4_0 \,|\, 5_0\right)^2}{E_5 - E_4} + 2e^2 \frac{\left(5_0 \,|\, 6_0\right)^2}{E_6 - E_5} + 8e^2 \sum_{m=3}^{4} \frac{\left(4_m \,|\, 5_m\right)^2}{E_5 - E_4} + 8e^2 \sum_{m=1}^{2} \frac{\left(5_m \,|\, 6_m\right)^2}{E_6 - E_5} \tag{S14}$$

2.2. Polarizability of the first excited state of C_{60}^{+}

The polarizability of the C_{60}^{+} molecule in the first excited state comes from the coupling between this state and the ground and all other excited states resulting from single excitations from the first excited state.

The Slater determinant contributing to this polarizability are:

(i) The ground state, $4_0 \leftarrow 5_0$ excitation $\left(\Delta l = -1; m = m' = 0\right)$ with one open shell:

$$\Psi_0 = \Psi'_{4 \leftarrow 5} = \left|\ldots 4_0 \overline{4_0} 5_0\right| \tag{S15}$$

The corresponding contribution to the first excited state polarizability is

$$\alpha_1^{0} = 2e^2 \frac{\left|\left\langle \Psi_1 \,|\, z \,|\, \Psi_0 \right\rangle\right|^2}{E_4 - E_5} = -2e^2 \frac{\left(4_0 \,|\, 5_0\right)^2}{E_5 - E_4} \tag{S16}$$

(ii) The $3_0 \to 4_0$ excitation $\left(\Delta l = l; m = m' = 0\right)$ with one open shell:

$$\Psi'_{3 \to 4} = \left|\ldots 3_0 4_0 \overline{4_0}\right| \tag{S17}$$

The corresponding contribution to the first excited state polarizability is

$$\alpha_1^{3_0\to 4_0} = 2e^2 \frac{\left(3_0 \mid 4_0\right)^2}{E_4 - E_3} \tag{S18}$$

(iii) The $5_0 \to 6_0$ excitation $\left(\Delta l = l; m = m' = 0\right)$ with three open shells. This configuration has two doublets.

$$\Psi'^{a}_{5_0\to 6_0} = \frac{1}{\sqrt{2}}\left(\left|\ldots 4_0 5_0 \overline{6_0}\right| - \left|\ldots 4_0 \overline{5_0} 6_0\right|\right) \tag{S19}$$

$$\Psi'^{b}_{5_0\to 6_0} = \frac{1}{\sqrt{6}}\left(\left|\ldots 4_0 5_0 \overline{6_0}\right| + \left|\ldots 4_0 \overline{5_0} 6_0\right| - 2\left|\ldots \overline{4_0} 5_0 6_0\right|\right) \tag{S20}$$

The corresponding contribution to the first excited state polarizability is

$$\alpha_1^{5_0\to 6_0} = 4e^2 \frac{\left(5_0 \mid 6_0\right)^2}{E_6 - E_5} \tag{S21}$$

(iv) The $4_m \to 5_{-m}$ excitation $\left(\Delta l = 1; m = -m' = \pm 3, \pm 4\right)$ with three open shells. This configuration has two doublets:

$$\Psi'^{a}_{4_m\to 5_{-m}} = \frac{1}{\sqrt{2}}\left(\left|\ldots 4_0 4_m \overline{5_{-m}}\right| - \left|\ldots 4_0 \overline{4_m} 5_{-m}\right|\right) \tag{S22}$$

$$\Psi'^{b}_{4_m\to 5_{-m}} = \frac{1}{\sqrt{6}}\left(\left|\ldots 4_0 4_m \overline{5_{-m}}\right| + \left|\ldots 4_0 \overline{4_m} 5_{-m}\right| - 2\left|\ldots \overline{4_0} 4_m 5_{-m}\right|\right) \tag{S23}$$

The corresponding contribution to the first excited state polarizability is

$$\alpha_1^{4_m\to 5_{-m}} = 8e^2 \sum_{m=3}^{4} \frac{\left(4_m \mid 5_m\right)^2}{E_5 - E_4} \tag{S24}$$

(v) The $5_m \to 6_{-m}$ excitation $\left(\Delta l = 1; m = -m' = \pm 1, \pm 2\right)$ with three open shells. This configuration has two doublets:

$$\Psi'^{a}_{5_m\to 6_{-m}} = \frac{1}{\sqrt{2}}\left(\left|\ldots 5_0 5_m \overline{6_{-m}}\right| - \left|\ldots 5_0 \overline{5_m} 6_{-m}\right|\right) \tag{S25}$$

$$\Psi'^{b}_{5_m\to 6_{-m}} = \frac{1}{\sqrt{6}}\left(\left|\ldots 5_0 5_m \overline{6_{-m}}\right| + \left|\ldots 5_0 \overline{5_m} 6_{-m}\right| - 2\left|\ldots \overline{5_0} 5_m 6_{-m}\right|\right) \tag{S26}$$

The corresponding contribution to the first excited state polarizability is

$$\alpha_0^{5_m \to 6_{-m}} = 8e^2 \sum_{m=1}^{2} \frac{\left(5_m \mid 6_m\right)^2}{E_6 - E_5} \tag{S27}$$

Collecting all these contributions, the polarizability of C_{60}^+ in the first excited state is given by

$$\begin{aligned} \alpha_1 &= -2e^2 \frac{\left(4_0 \mid 5_0\right)^2}{E_5 - E_4} + 2e^2 \frac{\left(3_0 \mid 4_0\right)^2}{E_4 - E_3} + 4e^2 \frac{\left(5_0 \mid 6_0\right)^2}{E_6 - E_5} \\ &+ 8e^2 \sum_{m=3}^{4} \frac{\left(4_m \mid 5_m\right)^2}{E_5 - E_4} + 8e^2 \sum_{m=1}^{2} \frac{\left(5_m \mid 6_m\right)^2}{E_6 - E_5} \end{aligned} \tag{S28}$$

Supplementary References

1. Ralser, S., Postler, J., Harnisch, M., Ellis, A. M. & Scheier, P. Extracting cluster distributions from mass spectra: IsotopeFit. *Int. J. Mass Spectrom.* **379**, 194-199 (2015).

2. Campbell, E. K., Holz, M., Gerlich, D. & Maier, J. P. Laboratory confirmation of C_{60}^+ as the carrier of two diffuse interstellar bands. *Nature* **523**, 322-323 (2015).
